# Case Report: Lung transplantation for rare anti-MDA5–positive rapidly progressive interstitial lung disease: two cases and a narrative review

**DOI:** 10.3389/fimmu.2026.1817318

**Published:** 2026-04-22

**Authors:** Qing Zhang, Xiao-dong Li, Yang Li

**Affiliations:** 1 Department of Surgery, The First Hospital of Jilin University, Changchun, China; 2 Department of Thoracic Surgery, Organ Transplantation Center, The First Hospital of Jilin University, Changchun, China

**Keywords:** anti-MDA5 antibody, dermatomyositis, lung transplantation, rapidly progressive interstitial lung disease, extracorporeal membrane oxygenation

## Abstract

Lung transplantation is a definitive lifesaving option for end-stage lung disease; however, its role in rapidly progressive interstitial lung disease (RP-ILD) associated with anti–melanoma differentiation-associated gene 5 (anti-MDA5) antibodies remains debated. We retrospectively analyzed two anti-MDA5–positive RP-ILD (MDA5^+^ RP-ILD) patients who underwent bilateral lung transplantation and synthesized pertinent literature to evaluate clinical value and the impact of therapeutic interventions. Both patients recovered postoperatively and were discharged after symptomatic management and supportive care. One patient subsequently developed cerebral infarction and died approximately 8 months after transplantation, whereas the other remains clinically stable under regular follow-up. Given the limited efficacy of conventional therapies and the overall poor prognosis of anti-MDA5-positive dermatomyositis (MDA5^+^ DM) with RP-ILD, our observations-together with prior reports—support lung transplantation, with extracorporeal membrane oxygenation (ECMO) as a bridge when necessary, as a promising and reliable therapeutic strategy. Lung transplantation may offer definitive treatment in carefully selected patients, underscoring its important clinical value.

## Introduction

Clinically amyopathic dermatomyositis (CADM) is a distinct clinical subtype within the idiopathic inflammatory myopathies (IIM) and is reported more frequently in Asian populations, particularly in China and Japan (1). Patients typically show the cutaneous hallmarks of dermatomyositis, whereas skeletal muscle involvement is mild or even absent (2, 3). Prior to the identification of autoantibodies against melanoma differentiation-associated gene 5 (MDA5), many cases now recognized as MDA5^+^ DM were categorized as CADM; the discovery of MDA5 antibodies prompted a redefinition and renaming of this entity as MDA5^+^ DM (4).

The prognosis of MDA5^+^ DM largely hinges on the presence and severity of interstitial lung disease (ILD). Compared with classic dermatomyositis, MDA5^+^ DM is more frequently complicated by RP-ILD and portends a worse outcome (5). RP-ILD is commonly defined by progression to respiratory failure within three months from the onset of respiratory symptoms, or by marked radiologic deterioration within one month of dyspnea onset (6). Once RP-ILD develops, disease evolution is abrupt, effective disease-modifying options are limited, and mortality remains high despite conventional immunosuppressive therapy; indeed, mortality has been reported to exceed 50% within three months in CADM patients with RP-ILD (7).

Against this backdrop, the clinical role and timing of lung transplantation for MDA5^+^ DM–associated RP-ILD remain insufficiently characterized, with existing evidence primarily derived from case reports and small series. In 2024, two patients with MDA5^+^ DM complicated by RP-ILD were managed at the First Hospital of Jilin University and underwent sequential bilateral lung transplantation. One patient showed marked clinical improvement, whereas the other achieved recovery sufficient for discharge but subsequently experienced an adverse outcome during follow-up. Here, we retrospectively describe their clinical features, diagnostic work-up, imaging and laboratory findings, and therapeutic interventions, and we contextualize these observations within the current literature to appraise the potential value of lung transplantation in this high-risk population and the role of supportive strategies such as ECMO as a bridge to transplantation in MDA5^+^ DM–associated RP-ILD.

## Case presentation

### Case 1

A 55-year-old man was diagnosed with cutaneous lupus erythematosus at our Department of Rheumatology and Immunology one month before the current admission. After discharge, he was treated with regular oral methylprednisolone and hydroxychloroquine sulfate. One week prior to admission, the patient developed cough, sputum production, and progressively worsening dyspnea without obvious precipitating factors, accompanied by intermittent fever, with a maximum temperature of 38.6 °C. Anti-infective treatment at a local hospital was ineffective. Subsequent laboratory evaluation revealed positivity for MDA5 antibody, and MDA5^+^ DM complicated by RP-ILD was suspected. The patient was therefore transferred to the First Hospital of Jilin University on July 26, 2024, for further management.

**Figure 1:**
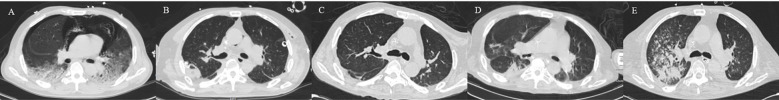
Serial chest CT images of Case 1. **(A)** Preoperative CT showing diffuse bilateral patchy and confluent high-density opacities with pneumothorax and pneumomediastinum. **(B)** CT at 7 days post-transplantation demonstrating marked improvement in pulmonary lesions. **(C)** CT at 3 months postoperatively showing relatively stable findings with minimal residual opacities. **(D)** CT at 6 months showing mild progression of fibrotic changes. **(E)** CT at 8 months showing increased patchy high-density opacities.

On admission, chest computed tomography (CT) (**Figure 1A**) demonstrated diffuse bilateral patchy and confluent high-density opacities with ill-defined margins, accompanied by bilateral pneumothorax. On this CT image, linear and curvilinear gas lucencies were present in the anterior mediastinum, consistent with pneumomediastinum. Arterial blood gas analysis revealed an arterial partial pressure of oxygen (PaO_2_) of 63 mmHg under a fraction of inspired oxygen (FiO_2_) of 60%, with a lactate level of 2.3 mmol/L. Serological testing showed IgG positivity for cytomegalovirus, Mycoplasma pneumoniae, and Chlamydia pneumoniae, indicating prior exposure rather than necessarily active infection. In addition, blood mNGS detected sequences corresponding to cytomegalovirus, Mycoplasma pneumoniae, Chlamydia pneumoniae, and Pneumocystis jirovecii, raising concern for possible concurrent infections in the clinical context. Serum ferritin was markedly elevated (2347.0 μg/L); however, serial reassessment of serum ferritin was not performed during the subsequent treatment course. Autoantibody testing demonstrated a high titer of anti-MDA5 antibody (1:300) and positivity for anti-Ro52 antibody (titer 1:10).

After admission, the patient was initially treated with methylprednisolone in combination with hydroxychloroquine sulfate. One week prior to transplantation, the therapeutic regimen was adjusted to include methylprednisolone (40 mg twice daily), intravenous immunoglobulin (25 g once daily), and tacrolimus (1 mg twice daily) for the management of dermatomyositis and for control of dermatomyositis-associated inflammation and peri-transplant immunosuppressive optimization.

Despite intensive comprehensive treatment, including broad-spectrum anti-infective therapy, immunosuppressive therapy, bronchodilators, and noninvasive ventilation, the patient’s respiratory function continued to deteriorate. After multidisciplinary team evaluation, the patient was considered eligible for lung transplantation, and sequential bilateral lung transplantation was performed under ECMO support.

Basiliximab was administered preoperatively to reduce the risk of acute rejection following transplantation. Postoperatively, the initial immunosuppressive regimen consisted of tacrolimus (1.5 mg twice daily) combined with methylprednisolone (40 mg once daily). At 12 hours after surgery, the tacrolimus trough concentration was 4.3 ng/mL, which was below the therapeutic range, suggesting a potentially increased risk of rejection. Considering the short duration of administration, the original immunosuppressive regimen was maintained.

On postoperative day 4, the tacrolimus trough level increased to 9.8 ng/mL. Concurrently, the total CD3^+^ T-cell count and the absolute number of CD8^+^ cytotoxic T cells showed a marked decline, indicating a reduced likelihood of acute rejection. A progressive increase in serum creatinine was observed following tacrolimus administration, reaching 163.6 μmol/L on postoperative day 4. Urinalysis revealed hematuria (25 red blood cells per high-power field), positive proteinuria, and an elevated epithelial cell count (116/μL). These findings were considered suggestive of tacrolimus-associated acute kidney injury.

Accordingly, the immunosuppressive regimen was adjusted to cyclosporine (125 mg twice daily) in combination with methylprednisolone (40 mg once daily), which was continued until discharge.

Chest CT obtained seven days after lung transplantation (**Figure 1B**) demonstrated marked improvement in pulmonary findings compared with preoperative imaging. The overall lung fields appeared clearer, without evidence of extensive or diffuse inflammatory exudative shadows. Comparison of pre- and postoperative chest CT scans suggested that lung transplantation may partially interrupt the progression of pulmonary fibrosis in this end-stage patient with anti-MDA5 antibody-positive RP-ILD, leading to a favorable clinical outcome. Repeat immunological testing on postoperative day 39 demonstrated seroconversion of anti-MDA5 antibodies to negative. Under close multidisciplinary collaboration, the patient recovered well and was discharged.

During the first three months of follow-up, chest CT (**Figure 1C**) findings in Case 1 remained relatively stable, showing only a small amount of subpleural linear opacities and patchy shadows in both lungs. At six months postoperatively, CT (**Figure 1D**) imaging revealed mildly increased fibrotic linear opacities along with a small number of patchy high-density lesions compared with previous examinations. By eight months after transplantation (the final CT assessment, **Figure 1E**), there was a marked increase in the extent and density of patchy high-attenuation opacities.

Throughout the follow-up period, the patient received immunosuppressive therapy with cyclosporine in combination with oral prednisone, with dosages adjusted according to clinical and follow-up findings. Subsequent telephone follow-up revealed that the patient died at another hospital due to sudden cerebral infarction.

**Figure 2:**
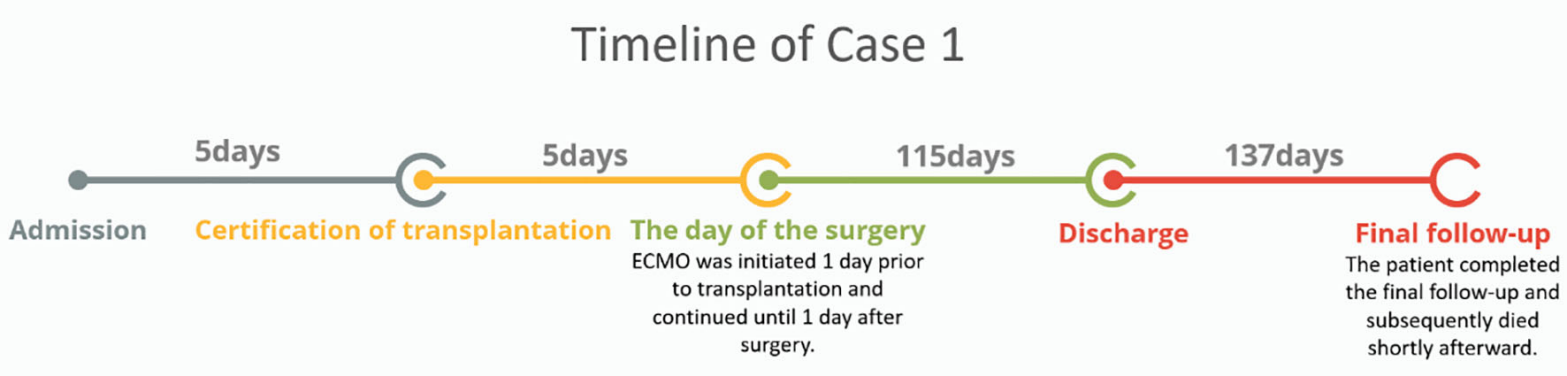
Timeline of Case 1. The patient was admitted, evaluated for transplantation after 5 days, underwent surgery 5 days later with ECMO support, was discharged on postoperative day 115, and followed up until death.

Timeline of Case 1: As shown in the timeline (**Figure 2**), Case 1 was deemed eligible for lung transplantation five days after admission, and the procedure was performed five days later. ECMO support was initiated one day prior to transplantation and continued until postoperative day 1. The patient met the discharge criteria on postoperative day 115 and was subsequently discharged. Regular follow-up was conducted after discharge, with the final follow-up completed 137 days from the discharge, after which the patient died shortly thereafter.

### Case 2

A 59-year-old man was admitted to our hospital. Approximately two weeks before admission, the patient developed cough with white mucoid sputum accompanied by fever, with a body temperature of approximately 38 °C, without obvious precipitating factors. He received symptomatic supportive treatment at a local hospital, including anti-infective therapy, expectorants, and bronchodilators; however, no significant clinical improvement was observed. Over the subsequent four days, both the frequency and severity of fever increased, with a peak temperature of 38.8 °C, and progressive dyspnea developed; therefore, the patient was transferred to our hospital for further management.

**Figure 3:**
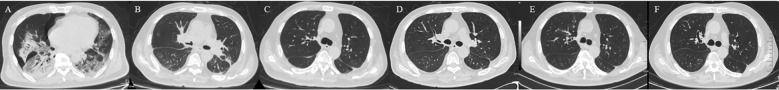
Serial chest CT images of Case 2. **(A)** Preoperative CT showing extensive bilateral ground-glass opacities and pneumothorax. **(B)** Postoperative CT demonstrating significant resolution of pulmonary lesions. **(C–F)** CT images at 3, 6, 9, and 19 months showing progressive improvement and stabilization of lung condition.

Repeat chest CT on admission (**Figure 3A**) revealed extensive bilateral ground-glass opacities, with spontaneous pneumothorax and marked progression compared with prior imaging. Arterial blood gas analysis demonstrated a PaO_2_ of 57 mmHg under a fraction of inspired oxygen (FiO_2_) of 50%. Laboratory evaluation revealed positive quantitative blood Epstein–Barr virus (EBV) nucleic acid testing, while metagenomic next-generation sequencing (mNGS) of bronchoalveolar lavage fluid confirmed the presence of *Enterococcus faecalis* sequences, along with a markedly elevated serum ferritin level (1787.0 μg/L). After 28 days of treatment, serum ferritin decreased to 1099.7 μg/L prior to transplantation; no postoperative measurements were performed. Myositis-specific antibody testing demonstrated a positive anti-MDA5 antibody with a titer of 1:39. Based on the clinical manifestations, radiological features, and immunological findings, the patient was diagnosed with MDA5^+^ RP-ILD. After admission, the patient received methylprednisolone (40 mg twice daily), intravenous immunoglobulin (25 g once daily), and tacrolimus (1 mg twice daily) for approximately 20 days prior to transplantation for the treatment of MDA5^+^ RP-ILD.

Aggressive anti-infective therapy, noninvasive ventilatory support, and immunosuppressive treatments were administered; nevertheless, the patient developed refractory dry cough, progressively worsening dyspnea, and a progressive decline in oxygen saturation. After comprehensive evaluation, ECMO was initiated, combined with supplemental oxygen via nasal cannula to maintain adequate oxygenation. After one week of ECMO support, the patient’s condition stabilized. Following multidisciplinary team discussion, sequential bilateral lung transplantation via bilateral posterolateral thoracotomy was performed under ECMO assistance. The cold ischemia times were 7.3 hours for the right lung and 10.0 hours for the left lung, respectively.

Similar to Case 1, basiliximab was administered preoperatively to reduce the risk of immune rejection following lung transplantation. Postoperatively, the patient received immunosuppressive therapy with methylprednisolone (40 mg once daily) and tacrolimus (2 mg daily).

On postoperative day 3, pleural fluid culture confirmed infection with carbapenem-resistant *Klebsiella pneumoniae*. Given the requirement for polymyxin-based antimicrobial therapy and the concern for cumulative nephrotoxicity, the immunosuppressive regimen was adjusted to prednisone (30 mg once daily) in combination with cyclosporine (100 mg twice daily), which was continued until discharge.

Postoperative chest CT (**Figure 3B**) demonstrated complete resolution of the preoperative bilateral consolidations and ground-glass opacities, with good lung re-expansion and clear lung fields, without evident inflammatory infiltration, indicating favorable recovery of pulmonary function. Repeat immunological testing on postoperative day 42 demonstrated seroconversion of anti-MDA5 antibodies to negative.

With multidisciplinary collaboration and comprehensive supportive care, the patient gradually recovered and was discharged, followed by regular outpatient follow-up. During follow-up, the patient developed refractory bacterial infections, intermittently complicated by fungal infections, as well as bronchial anastomotic stenosis, requiring periodic bronchoscopic interventions and adjustment of antimicrobial therapy based on microbiological findings. Overall follow-up demonstrated favorable postoperative recovery, with a significant improvement in quality of life within one year after transplantation compared with the preoperative status. The patient remains under ongoing follow-up.

In Case 2, chest CT findings remained stable at 3 (**Figure 3C**), 6 (**Figure 3D**), 9 (**Figure 3E**), and 19 months (**Figure 3F**). No recurrence of the preoperative extensive bilateral ground-glass opacities was observed. The subtle postoperative linear opacities and inflammatory exudative changes gradually resolved over time, with restoration of overall lung transparency. Only minimal residual mild fibrotic linear changes were noted, suggesting that the patient had entered a stable recovery phase.

During follow-up, the patient received immunosuppressive therapy with cyclosporine and mycophenolate mofetil, with dosages adjusted according to clinical and follow-up findings.

**Figure 4:**
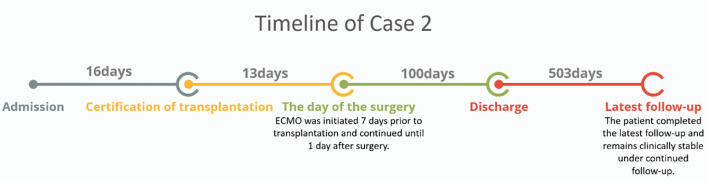
Timeline of Case 2. The patient was evaluated for transplantation 16 days after admission, underwent surgery 13 days later with ECMO support, was discharged on postoperative day 100, and remained stable during follow-up.

Timeline of case 2: As shown in the timeline (**Figure 4**), Case 2 was deemed eligible for lung transplantation 16 days after admission, and the procedure was performed 13 days later. ECMO support was initiated seven days prior to transplantation and continued until postoperative day 1. The patient met the discharge criteria on postoperative day 100 and was subsequently discharged. Regular follow-up was conducted after discharge, with the most recent follow-up completed 503 days from the discharge, during which the patient remained clinically stable.

## Discussion

### Pathogenesis

MDA5^+^ DM represents a distinct subtype of IIM, characterized by both the common clinical features of IIM and its own unique pathological mechanisms. Current research indicates that MDA5 is a member of the retinoic acid-inducible gene I-like receptors (RLRs) family and serves as a critical intracellular pattern recognition receptor (PRR) for detecting viral double-stranded RNA (dsRNA). MDA5 can be activated by viruses such as hepatitis A virus, Coxsackievirus B, enterovirus, rhinovirus, and other small RNA viruses, leading to the production of type I interferons (IFN-α and IFN-β) and various inflammatory cytokines, thereby initiating an antiviral immune response (8–10).

In contrast, persistent or excessive activation of the type I interferon (IFN) pathway is closely associated with the development of various autoimmune inflammatory disorders. RNA virus infections may upregulate MDA5 expression, leading to sustained activation of type I IFN signaling, which triggers the immunopathological cascade in MDA5^+^ DM (11). Under conditions of sustained MDA5 activation, the body’s immune tolerance is disrupted, prompting B lymphocytes to produce anti-MDA5 autoantibodies. These autoantibodies bind to MDA5, forming RNA immune complexes (RNA-ICs), which subsequently activate Toll-like receptor 7 (TLR7) on plasmacytoid dendritic cells (pDCs), stimulating persistent IFN-α secretion and establishing a positive feedback loop (“MDA5–ICs–TLR7–IFN-α”) (8, 12–14).

This positive feedback loop, along with immune complex formation, disrupts the body’s normal antiviral defense and activates macrophages and T lymphocytes via the “molecular mimicry” mechanism, leading to the release of pro-inflammatory cytokines (e.g., IL-8, IL-18, TNF-α, IFN-γ), thereby inducing an “inflammatory cytokine storm” (8, 11–13). The release of numerous inflammatory mediators directly damages alveolar epithelial cells and pulmonary microvascular endothelial cells, increasing vascular permeability, inducing alveolar edema and microthrombosis, and triggering characteristic cutaneous vasculitis manifestations (e.g., ulcers, palm papules) along with early pulmonary congestion and exudation, which explains the typical skin and lung lesions in MDA5^+^ DM patients (15–19).

It should be noted that elevated serum ferritin has been identified as an independent risk factor for the development of RP-ILD in patients with MDA5^+^ DM (20). When serum ferritin levels reach or exceed 823 ng/mL, the risk of RP-ILD increases significantly. On one hand, hyperferritinemia reflects the extent of macrophage activation and systemic inflammation; on the other, ferritin may exacerbate oxidative stress, thereby accelerating the onset and progression of pulmonary interstitial fibrosis and contributing to the deterioration of MDA5^+^ DM–associated lung lesions (1, 11, 18, 20, 21). In this study, the admission serum ferritin levels in both of our cases were markedly elevated, which is consistent with the aforementioned observations. To some extent, these findings may reflect the current activity of MDA5^+^ RP-ILD and suggest that the disease had progressed to, or was rapidly approaching, the end-stage fibrotic phase. Serum ferritin levels therefore represent an important and non-negligible biomarker for predicting disease progression and clinical outcomes in patients with MDA5^+^ RP-ILD.

### Treatment

The pathophysiological mechanisms underlying MDA5^+^DM with RP-ILD indicate that corticosteroids and immunosuppressive agents constitute the cornerstone of current therapeutic strategies (2, 21–23). Despite this aggressive immunosuppressive therapy, clinical responses are frequently suboptimal, and disease progression cannot be effectively controlled in a substantial proportion of patients. Furthermore, preventing the progression to end-stage pulmonary fibrosis remains a major clinical challenge in this population. Consequently, there is an urgent need for more effective therapeutic approaches capable of modifying the natural course and ultimate clinical outcomes of the disease.

Recent evidence has highlighted the potential role of Janus kinase (JAK) inhibitors in the management of anti-MDA5–positive RP-ILD. Compared with early case-based observations, emerging cohort-based and real-world studies have provided more robust support for the efficacy of JAK inhibitors, particularly tofacitinib, in improving disease control and survival in refractory patients. Nevertheless, despite these advances, a substantial proportion of patients still progress to end-stage lung disease, and lung transplantation remains a potential life-saving option for carefully selected individuals.

In this context, lung transplantation may represent a potential life-saving option for selected patients with refractory disease (24–27).

#### ECMO

In patients with MDA5^+^ DM complicated by RP-ILD, delayed or insufficient medical intervention often leads to extensive pulmonary fibrosis and severe respiratory failure. At this advanced stage, respiratory failure is frequently refractory to conventional mechanical ventilation and is accompanied by a marked reduction in PaO_2_ and oxygenation index (OI) (28).

Under such circumstances, ECMO serves a critical role in sustaining gas exchange and hemodynamic stability, functioning as a temporary bridge to subsequent therapeutic intervention or lung transplantation.

Evidence from previous literature and the two cases presented in this study indicates that, although lung transplantation remains the sole curative intervention for end-stage RP-ILD, sustaining life support during the donor-matching and waiting period constitutes a considerable clinical challenge. In our cases, rapid clinical deterioration was observed during the waiting period for transplantation. Preoperative ECMO support was initiated to maintain vital organ function, enabling both patients to successfully receive matched donor lungs and complete transplantation while remaining on ECMO (28, 29). A multicenter study conducted in France reported that all five patients with MDA5^+^ RP-ILD who underwent ECMO-bridged lung transplantation survived, demonstrating a significantly better outcome compared with those treated with ECMO alone. These findings further underscore the clinical value of ECMO as a bridging strategy prior to lung transplantation (30).

These findings emphasize the pivotal role of ECMO as a critical bridge-to-transplantation strategy, offering a practical means to prolong survival and enable successful lung transplantation in patients with end-stage RP-ILD.

#### Lung transplantation as a surgical treatment

In previous clinical practice for MDA5^+^ RP-ILD, forced vital capacity (FVC) has commonly been used as a key parameter for assessing disease progression and predicting prognosis. In the context of lung transplantation, a more refined approach to patient stratification and risk assessment is required. A previously reported prognostic model indicated that patients with a baseline FVC <50% had an approximate 6-month mortality of 50%, whereas those unable to tolerate pulmonary function testing exhibited a mortality rate exceeding 90% within the same period (31).

Notably, both patients in our study developed severe respiratory failure that could not be corrected by conventional respiratory support prior to transplantation, rendering them unable to undergo pulmonary function testing. This observation suggests that the inability to tolerate pulmonary function assessment should not be regarded merely as missing data, but rather as an important indicator of disease severity that warrants incorporation into clinical stratification and therapeutic decision-making.

Apart from FVC and oxygenation index (OI), radiological features also merit careful consideration. In our cases, both patients exhibited spontaneous pneumothorax on CT imaging, with Case 1 further complicated by pneumomediastinum. Previous studies have reported that the presence of spontaneous pneumothorax and/or pneumomediastinum during the acute phase of MDA5^+^ RP-ILD may serve as predictors of increased mortality (32).

Taken together, these findings suggest that both patients were at an extremely high risk of adverse clinical outcomes, underscoring the urgent need for interventions capable of halting disease progression. Under such circumstances, lung transplantation may represent a reasonable and potentially life-saving option in the current cases. The evidence from the aforementioned studies further provides a valuable theoretical basis for patient selection and clinical decision-making in the context of lung transplantation for MDA5^+^ RP-ILD.

By replacing diseased pulmonary tissue, lung transplantation directly enhances ventilation and gas exchange, markedly relieves symptoms of respiratory failure, and prolongs overall survival. Although lung transplantation remains the final therapeutic option for end-stage RP-ILD, the number of documented cases in patients with MDA5^+^ DM remains small, and additional clinical studies are required to confirm its efficacy and long-term outcomes. Nevertheless, existing case reports indicate that lung transplantation may provide substantial survival benefits in this patient population. In one reported cohort, 10 of 11 patients (90%) with MDA5^+^ DM–associated RP-ILD who underwent lung transplantation were successfully discharged, achieving a one-year survival rate of 82% (28). In another report, three out of four patients with MDA5^+^ RP-ILD who underwent lung transplantation survived during long-term follow-up, further supporting the important role and clinical value of lung transplantation in prolonging survival in this patient population (33).

From the perspective of postoperative complications, patients with MDA5^+^ RP-ILD share several common complications observed after conventional lung transplantation; however, this specific population also exhibits distinct clinical characteristics. Notably, many patients are already in a state of severe systemic inflammation prior to transplantation, often accompanied by a “cytokine storm.” This pre-existing inflammatory milieu may persist into the early postoperative period and compromise graft stability. It can directly damage alveolar epithelial cells and pulmonary microvascular endothelial cells, thereby contributing to the development of primary graft dysfunction (PGD), which remains one of the leading causes of early postoperative mortality.

Moreover, immune rejection represents an unavoidable concern following lung transplantation. As discussed above, in the “MDA5–ICs–TLR7–IFN-α” positive feedback loop characteristic of MDA5^+^ RP-ILD, elevated type I interferon (IFN-I) not only sustains this pathogenic circuit but may also promote DC activation, thereby participating in both acute and chronic allograft rejection. This mechanism may increase the complexity of postoperative immunosuppressive management in this patient population (34).

Another unique complication is the recurrence or flare of DM. Currently, literature focusing specifically on DM recurrence after lung transplantation in MDA5^+^ RP-ILD remains limited, consisting mainly of case reports and small case series. In one previously reported case, only a single patient experienced DM flare and subsequently died (28). In contrast, a broader cohort study of IIM-associated ILD transplantation reported a recurrence rate of approximately 8%, predominantly involving the skin and muscle, without direct involvement of the transplanted lung (35).

Based on these observations, we speculate that this phenomenon may be related to the disruption of the MDA5^+^ DM-associated positive feedback loop. If the diseased lung serves as a major source of persistent RNA antigens and tissue injury signals, its removal through transplantation may substantially attenuate this pathogenic circuit (13). This hypothesis may also help explain the gradual decline or even seroconversion to negativity of anti-MDA5 antibody titers observed after transplantation in previous studies.

Furthermore, compared with preoperative treatment, postoperative immunosuppressive regimens are typically more sustained, stable, and multidimensional. This may partly account for the apparent discrepancy whereby patients exhibit refractoriness to intensive immunosuppressive therapy before transplantation but achieve disease remission thereafter (27). Nevertheless, the precise mechanisms underlying the decline in anti-MDA5 antibody titers and the relatively low incidence of DM flare after transplantation remain unclear, and further mechanistic studies are warranted to elucidate these processes.

#### Clinical observation and case value

Both patients described in this study achieved successful postoperative recovery and were discharged, although their subsequent clinical courses diverged. Case 1 completed the final follow-up 252 days after the surgery, but subsequently died of a sudden cerebrovascular event at another institution. Case 2 demonstrated favorable recovery and remained clinically stable at the most recent follow-up, completed 542 days after surgery.

In our cases, patients presented with fever and multiple concurrent infections at the time of hospitalization. Based on previous reports, the susceptibility to infections in patients with MDA5^+^ RP-ILD may be attributed to several underlying mechanisms.

Alterations associated with anti-MDA5 antibodies may impair antiviral and antifungal immune responses, which could partly explain the increased risk of opportunistic infections in this population (36). Despite sustained activation of the interferon (IFN) pathway driven by the previously described positive feedback loop, excessive IFN signaling does not necessarily translate into effective antimicrobial defense, but is more likely to result in immune dysregulation, increased cellular apoptosis, and tissue injury (37).

In this immunological setting, natural killer (NK) cells are often reduced in proportion and exhibit enhanced apoptosis, while neutrophils remain in a state of aberrant activation, predisposing them to excessive neutrophil extracellular trap formation (NETosis). These alterations collectively impair innate immune and cytotoxic clearance capacity, leading to increased inflammation and tissue damage rather than efficient pathogen elimination (38).

Macrophage overactivation and the associated cytokine storm during disease progression further damage alveolar epithelial cells and disrupt the integrity of the pulmonary barrier, thereby increasing susceptibility to secondary infections (39–41).

Notably, patients with MDA5^+^ DM complicated by RP-ILD almost invariably present with elevated anti-MDA5 antibody levels. Several case reports have indicated that anti-MDA5 antibody titers should ideally be reduced or converted to seronegative status before lung transplantation. In these studies, patients achieving seronegativity prior to transplantation did not experience postoperative relapse, whereas those with persistently elevated preoperative titers demonstrated relapse rates approaching 50%. Therefore, preoperative surveillance and regulation of anti-MDA5 antibody levels with intensified corticosteroid and immunosuppressive therapy should be regarded as integral elements of transplant assessment and perioperative management (42).

Furthermore, both patients gradually became seronegative for autoantibodies, particularly anti-MDA5, after lung transplantation. These observations suggest that lung transplantation not only mitigates life-threatening complications such as respiratory failure but may also contribute to the resolution of underlying immune dysregulation.

Previous reports have indicated that a subset of patients may exhibit persistently elevated anti-MDA5 antibody levels after lung transplantation, a finding that is generally associated with poor prognosis. This observation is of particular interest. Given that the primary aim of lung transplantation is to replace the structurally damaged lung, postoperative antibody dynamics likely reflect the combined effects of preoperative immunosuppression and postoperative immune control (28).

Insufficient suppression of immune activity before or after transplantation may contribute to persistently elevated or increased anti-MDA5 antibody titers. In contrast, in our cases, anti-MDA5 antibody levels gradually declined and eventually became negative following sustained postoperative immunosuppressive therapy. This finding may, to some extent, support the hypothesis that the diseased lung serves as a major source of persistent RNA antigens and tissue injury signals.

These observations highlight the importance of reducing anti-MDA5 antibody titers as much as possible prior to transplantation to minimize the risk of ongoing disease activity after surgery, which is consistent with previous reports.

It should also be noted that MDA5 functions as a cytosolic sensor of viral RNA. Postoperative factors such as infection, ischemia–reperfusion injury, and systemic inflammatory responses may therefore reactivate this pathway and potentially trigger disease flare. In this context, perioperative and postoperative management in patients with MDA5^+^ RP-ILD may require stricter monitoring and more rigorous immunological control than in conventional lung transplant recipients.

These findings indicate that lung transplantation may enable long-term survival even in patients with advanced MDA5^+^ DM complicated by RP-ILD. These results provide novel insights and potential therapeutic strategies for the comprehensive management of MDA5^+^ DM–associated RP-ILD, which may help improve the historically poor prognosis of this condition.

Although lung transplantation offers substantial clinical benefits for patients with advanced MDA5^+^ DM complicated by RP-ILD, its implementation remains constrained by multiple challenges, foremost among them the complexity of donor–recipient matching. Owing to sociocultural and ethical influences, public awareness and acceptance of organ donation remain limited in many regions, leading to a persistent shortage of suitable donors. As a consequence, numerous patients with terminal RP-ILD experience fatal outcomes primarily because of the scarcity of donor lungs. Therefore, optimizing donor acquisition and matching strategies constitutes a critical bottleneck hindering the advancement of lung transplantation in MDA5^+^ DM–associated RP-ILD.

Of particular note, in one of the previously described cases, a patient with MDA5^+^ DM complicated by RP-ILD exhibited significant short-term pulmonary improvement following surgical intervention but later developed sudden cerebral infarction and unexpected death during postoperative recovery. Based on the clinical course, the cerebral infarction was suspected to be closely associated with the underlying immunopathological mechanisms of MDA5^+^ DM. The cytokine storm induced by the “MDA5–ICs–TLR7–IFN-α” positive feedback loop may have led to microvascular endothelial injury, increased vascular permeability, and microthrombus formation, thereby acting as a potential precipitating factor for cerebral infarction.

Furthermore, previous studies have demonstrated that mild activation of IFN-I signaling pathway enhances ischemic tolerance in cerebral tissue via the IRF3/7 axis. However, under conditions of acute and severe ischemia, excessive activation of the same pathway disrupts the integrity of the blood–brain barrier (BBB) and aggravates secondary ischemic brain injury, acting as an “amplifier” of neuroinflammation (43, 44). This mechanism may partly account for the rapid clinical deterioration observed in this patient after the onset of cerebral infarction.

Nevertheless, in the absence of autopsy findings and etiological confirmation, the precise cause of cerebral infarction remains undetermined. Current evidence regarding the occurrence of cerebral infarction in patients with MDA5^+^ DM complicated by RP-ILD is scarce, and no definitive causal association has been established. Future studies aimed at elucidating the relationship between the immune-inflammatory state of MDA5^+^ DM and cerebrovascular events are warranted, as they would offer important insights into the management, rehabilitation, and prognostic assessment of affected patients.

#### Limitations

Several limitations of this study should be acknowledged. First, this report is a retrospective two-case study accompanied by a narrative review, and its main purpose was to summarize clinical experience and lessons derived from real-world practice rather than to provide systematic evidence for treatment guidance. Given the very small sample size, the conclusions drawn from these cases are necessarily limited and should be interpreted with caution. In addition, our review of the literature on lung transplantation in patients with MDA5^+^ DM was not fully systematic; therefore, the available evidence may not have been comprehensively captured, and some relevant reports may have been missed.

Second, the monitoring of several clinically important biomarkers was incomplete. In particular, ferritin, which may provide useful information regarding disease activity, progression, and prognosis, was not serially assessed before and after transplantation. The lack of such data limited our ability to more comprehensively evaluate disease course and peri-transplant risk. This also highlights the importance of systematic biomarker monitoring in the management of such patients.

Third, because of financial constraints and barriers to follow-up accessibility, regular long-term pulmonary function testing and continuous immunological surveillance were not consistently performed after transplantation. This limited our ability to fully assess postoperative immune status and long-term graft and disease outcomes. Although previous reports have suggested that the incidence of post-transplant DM flare may be relatively low in this population, continued immunological monitoring remains essential for determining whether dermatomyositis remains quiescent or shows signs of recurrence or progression.

Despite these limitations, the present two cases provided our center with a more comprehensive understanding of the diagnosis, perioperative management, and follow-up of patients with MDA5^+^ DM-associated RP-ILD. They also further support the potential role of lung transplantation as a rescue strategy for improving clinical outcomes in carefully selected patients.

## Conclusion

MDA5^+^ DM complicated by RP-ILD is distinguished by the swift onset of pulmonary fibrosis within a limited timeframe, frequently culminating in severe respiratory failure. In patients with less advanced disease, the principal therapeutic objective is the prompt suppression of inflammation via immunosuppressive therapy, aiming to attenuate the cytokine storm and slow the progression of RP-ILD.

For patients refractory to first-line therapy or those who progress to terminal-stage disease, a multidimensional approach integrating salvage immunosuppressive therapy, antifibrotic agents, and advanced respiratory support may help stabilize the condition and create a therapeutic window for lung transplantation. Lung transplantation, in turn, can markedly improve prognosis in end-stage cases, allowing selected patients to achieve long-term survival and, in rare instances, sustained clinical remission. Given the limited availability of lung transplantation, a more cautious approach to patient selection is warranted. Factors such as oxygenation index (OI), forced vital capacity (FVC), the occurrence of spontaneous pneumomediastinum and/or pneumothorax during the acute phase, as well as the inability to tolerate pulmonary function testing in advanced disease, should all be incorporated into the assessment.

Although clinical experience with lung transplantation in MDA5^+^ DM–associated RP-ILD remains limited, accumulating evidence suggests that the procedure confers substantial therapeutic and survival advantages for patients with otherwise fatal disease. Nevertheless, further systematic investigations are warranted to validate the long-term efficacy, safety, and optimal perioperative management strategies of lung transplantation in this distinct and high-risk patient cohort.

## Data Availability

The original contributions presented in the study are included in the article/supplementary material. Further inquiries can be directed to the corresponding author.
